# The complete chloroplast genome sequence of *Durio oxleyanus* (Malvaceae) and its phylogenetic position

**DOI:** 10.1080/23802359.2022.2123256

**Published:** 2022-09-23

**Authors:** Xue Jing Wong, Douglas Law, Zheng-Feng Wang, Shiamala Devi Ramaiya, Shiou Yih Lee

**Affiliations:** a Faculty of Health and Life Sciences, INTI International University, Nilai, Malaysia; b Guangdong Provincial Key Laboratory of Applied Botany, South China Botanical Garden, Chinese Academy of Sciences, Guangzhou, China; c Department of Crop Science, Faculty of Agricultural and Forestry Sciences, Universiti Putra Malaysia Bintulu Sarawak Campus, Bintulu, Malaysia

**Keywords:** Durian, Durioneae, genomic resource, next-generation sequencing, phylogenomics

## Abstract

*Durio oxleyanus* (Griff) of Malvaceae is considered a natural heritage by the countries that produce it, including Peninsular Malaysia, Sumatra, and Borneo. Even though the species is regarded as a commercially valuable fruit, cultivation of this species is uncommon. The dwindling population of this species in the wild has put its survival in jeopardy. Conservation efforts are required for this species, which are limited. In this study, the complete chloroplast (cp) genome of *D. oxleyanus* was assembled and characterized as a genomic resource for conservation programs. The complete cp genome size was 164,831 bp in length, with a pair of inverted repeats of 23,782 bp each, separating the 96,446-bp large and the 20,823-bp small single copies. A total of 135 genes were predicted, which consisted of 90 protein-coding, 37 tRNA, and eight rRNA genes. The overall GC content was 35.8%. The phylogenetic analysis based on the maximum-likelihood and Bayesian inference methods revealed that *D. oxleyanus* is closely related to *D. zibethinus*. The genomic data obtained will be useful for future studies of Malvaceae’s phylogenetics and evolution.

*Durio oxleyanus* Griff. 1845 of Malvaceae, is native to the tropics and is primarily found in the Southeast Asia region. The fruit of *D. oxleyanus*, like that of its sister species, *D. zibethinus* L. is edible (Lim 2012). When compared to other closely related species, *D. oxleyanus* can be recognized by its curvy and thorn fruit (Figure 1(A)) as well as its medium to large, oblong, leathery leaf that is densely stellate-hairy with scales along midrib, secondary veins and margin, abaxially (Figure 1(B)). However, *D. oxleyanus* is not widely cultivated, and the fruits are primarily sourced from the wild. Unfortunately, rapid urbanization has fragmented the forests, endangering the species’ survival in the wild (Munawaroh et al. 2020). Events of genetic erosion in *D. oxleyanus* have been reported due to a lack of attention to its genetic conservation (Brown 1997; Abdul Shukor et al. 2013). Recent work on the phylogenetic analysis of *Durio* was reconstructed based on the nuclear DNA internal transcribed spacer (ITS) sequences, which included at least 16 species of *Durio* species. However, the ITS sequence was not able to resolve the relationship at species level due to insufficient nucleotide variation that could delineate the closely related species (Nyffeler and Baum 2001). Chloroplast (cp) phylogenomics is an effective approach to analyze phylogenetic relationships among complicated plant species. To date, only two cp genomes of the *Durio* species have been characterized, in which both were derived from *D. zibethinus* (Cheon et al. 2017; Shearman et al. 2020). For a potential commercially driven fruit species, genetic studies on *D. oxleyanus* are generally lacking; hence, research on *D. oxleyanus* should be hastened. In this study, we sequenced and characterized the complete cp genome sequence of *D. oxleyanus* to serve as a valuable genomic resource for conservation actions.

**Figure 1.**
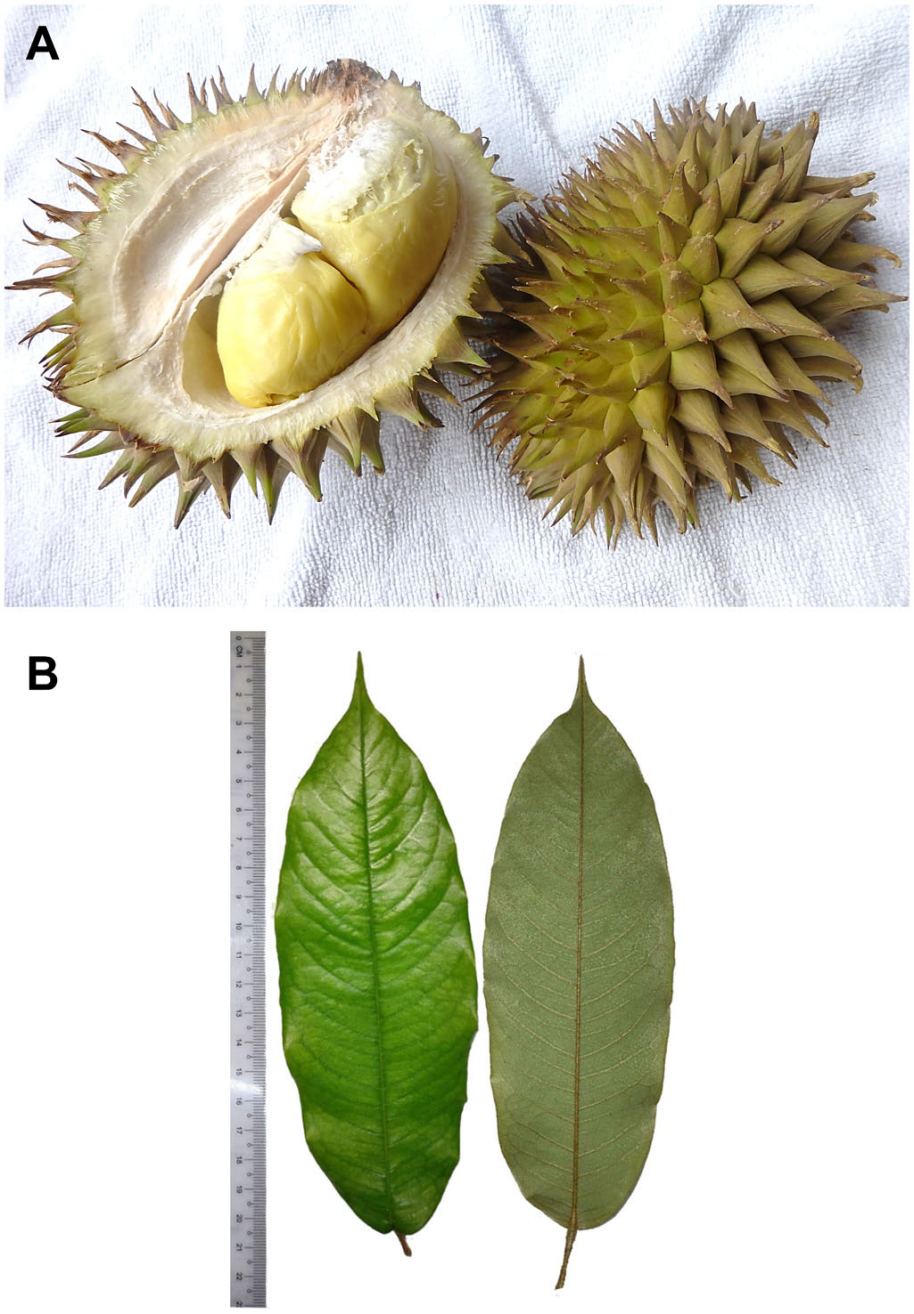
Morphological characteristics of *Durio oxleyanus*. (A) Fruit and (B) leaf.

Fresh leaves of *D. oxleyanus* were obtained from seedlings collected from the natural populations in Hulu Langat, Selangor (3°12′27″, 101°53′7″). The silica-dried leaf sample is catalogued as LSY1102 and was kept in the Molecular Biology Lab of INTI International University (https://newinti.edu.my, Dr. Lee Shiou Yih, shiouyih.lee@newinti.edu.my). Total genomic DNA was extracted using the FavorPrep™ Plant Genomic DNA Extraction Mini Kit (Favorgen, Taiwan, China), according to the manufacturer’s instructions. Preparation of a genomic library with an insert size of approximately 350 bp was conducted using the TruSeq DNA Sample Prep Kit (Illumina, San Diego, CA). Next-generation sequencing was carried out on the Illumina Novaseq (Illumina, San Diego, CA) platform, which generated 150 bp paired-ended raw reads. Genome assembly was implemented in Geneious Prime v2022.0.2 (Kearse et al. 2012) using the ‘map to reference’ function, with medium-low sensitivity, default settings, and five iterations applied. The complete *D. zibethinus* cp genome (GenBank accession number: MG138151) was selected as the reference genome. Gene annotation was performed using GeSeq v2.03 (Tillich et al. 2017) with the default parameters; the complete cp genomes of *D. zibethinus* (GenBank accession number: MG138151) and *Reevesia thyrsoidea* (GenBank accession number: MH939148) were chosen as annotation references. The output was manually checked for errors.

The complete *D. oxleyanus* cp genome sequence (GenBank accession number: ON653424) was 164,831 bp in length, containing four distinct regions, which included a large single-copy (96,446 bp), a small single-copy (20,823 bp), as well as a pair of inverted repeats (each 23,782 bp) (Figure 2). A total of 135 genes, consisting of 90 protein-coding, 37 tRNA genes, and eight rRNA genes, were predicted. The overall GC content was 35.8%.

**Figure 2.**
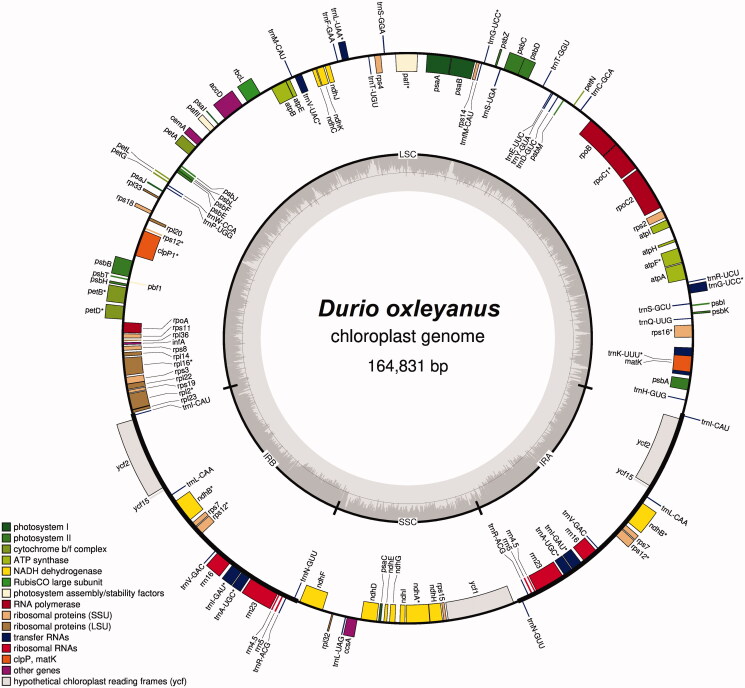
Chloroplast genome map of *Durio oxleyanus*. Genes on inside of map are transcribed in clockwise direction; genes on outside of map are transcribed in counter clockwise direction.

To ascertain the phylogenetic relationship and molecular placement of *D. oxleyanus* within Malvaceae, a phylogenetic reconstruction based on the 79 shared CDS sequences of the complete cp genome was carried out using 11 species. Two species, *Aquilaria sinensis* (Thymelaeaceae; GenBank accession number: MN720647; Deng et al. 2020) and *Vatica mangachapoi* (Dipterocarpaceae; GenBank accession number: MH716496; Wang et al. 2018) were included as outgroups. The CDS sequences were aligned using MAFFT 7.470 (Katoh and Standley 2013) and concatenated prior to phylogenetic analysis. Phylogenetic analysis was conducted using maximum-likelihood (ML) and Bayesian inference (BI) methods via RAxML v8 (Stamatakis 2014) and MrBayes v3.2 (Ronquist et al. 2012) pipelines available in the CIPRES Science Gateway (Miller et al. 2010). For ML, the tree was constructed using the general-time reversible (GTR) with gamma distribution (+G) (=GTR + G) nucleotide substitution model, coupled with 1000 bootstrap replicates. Markov chain Monte Carlo with 2,000,000 generations was used for BI, and sampling was taken every 100 cycles.

Both the ML and BI trees revealed the same topology, the trees were merged, and only the ML tree was displayed (Figure 3). The Malvaceae phylogenetic tree was well-resolved; bootstrap support (BS) and posterior probabilities (PPs) for each branch node were greater than 75% and 0.95, respectively. *Durio oxleyanus* was closely related to *D. zibethinus*; the divergence between *D. oxleyanus* and *D. zibethinus* is well supported with strong bootstrap value (BS = 100%, PP = 1.0). *Durio oxleyanus* was placed under Helicteroideae, along with two other species of *Reevesia*. The phylogenetic tree reconstructed using the cp genome sequences successfully revealed the relationship and molecular placement of *D. oxleyanus* with *D. zibethinus*, which were not able to be achieved when using the ITS sequences (Nyffeler and Baum 2001). From this study, the cp genome sequence seems to be potentially useful for the phylogenetic analysis of *Durio*, which might be able to facilitate the delimitation of the species. The genomic data obtained from this study will be beneficial for *D. oxleyanus* genetic conservation planning and future studies on *Durio* phylogenomic inferences and evolutionary genetics.

**Figure 3.**
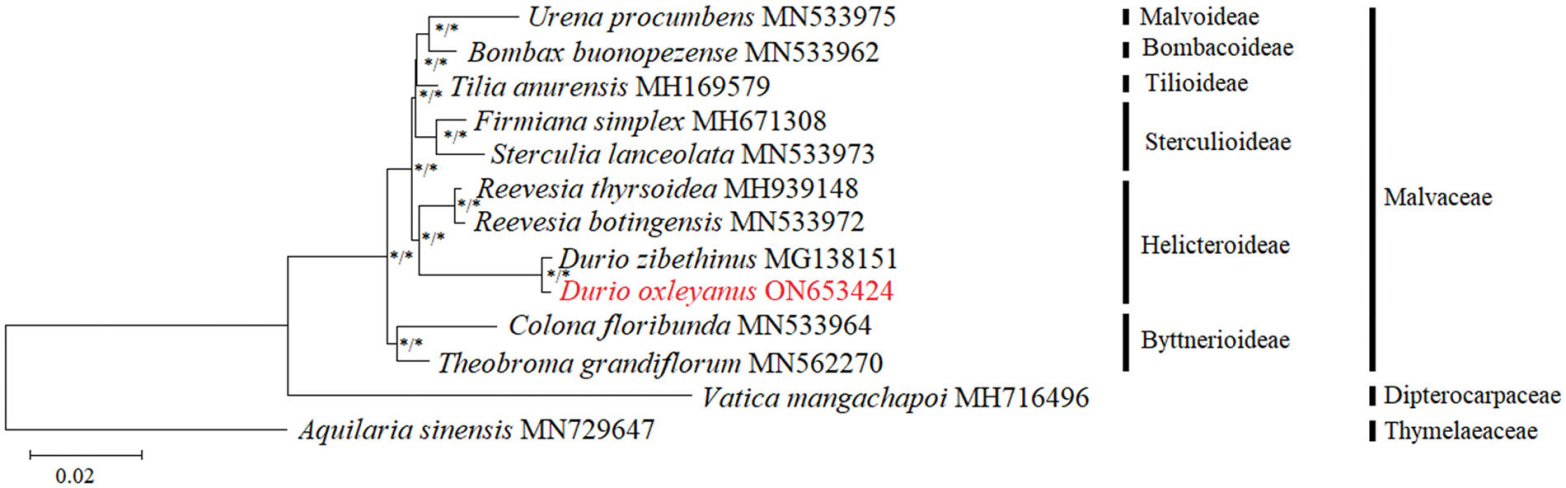
Phylogenetic tree based on the chloroplast genome sequence of 11 selected taxa of Malvaceae, with *Aquilaria sinensis* and *Vatica mangachapoi* included as outgroups. Shown next to the nodes are the bootstrap support (BS) and posterior probability (PP) values, in which strong branch support (BS ≥75%, left; PP ≥0.95, right) is indicated with an asterisk (*).

## Data Availability

The genome sequence data that support the findings of this study are openly available in GenBank of NCBI at http://www.ncbi.nlm.nih.gov under the accession number ON653424. The associated BioProject, SRA, and BioSample numbers are PRJNA845000, SRR19529291, and SAMN28853747, respectively.
